# Synergistic activity of troxacitabine (Troxatyl™) and gemcitabine in pancreatic cancer

**DOI:** 10.1186/1471-2407-7-121

**Published:** 2007-07-03

**Authors:** Vijaya L Damaraju, David Y Bouffard, Clarence KW Wong, Marilyn L Clarke, John R Mackey, Lorraine Leblond, Carol E Cass, Mike Grey, Henriette Gourdeau

**Affiliations:** 1 Department of Oncology, University of Alberta, and Cross Cancer Institute, Edmonton, Alberta T6G 1Z2, Canada; 2 Shire BioChem Inc., Ville St-Laurent, Québec H4S 2C9, Canada; 3 GTI Inc., Suite 200, 20925 Crossroads Circle, Waukesha, WI 53186-4054, USA; 4 SGX Pharmaceuticals Inc., 10505 Roselle Street, San Diego, CA 92121, USA

## Abstract

**Background:**

Gemcitabine, a deoxycytidine nucleoside analog, is the current standard chemotherapy used as first-line treatment for patients with locally advanced or metastatic cancer of the pancreas, and extends life survival by 5.7 months. Advanced pancreatic cancer thus remains a highly unmet medical need and new therapeutic agents are required for this patient population. Troxacitabine (Troxatyl™) is the first unnatural L-nucleoside analog to show potent preclinical antitumor activity and is currently under clinical investigation. Troxacitabine was recently evaluated as a first-line therapy in 54 patients with advanced adenocarcinoma of the pancreas and gave comparable overall results to those reported with gemcitabine in recently published randomized trials.

**Methods:**

The human pancreatic adenocarcinoma cell lines, AsPC-1, Capan-2, MIA PaCa-2 and Panc-1, were exposed to troxacitabine or gemcitabine alone or in combination, for 72 h, and the effects on cell growth were determined by electronic particle counting. Synergistic efficacy was determined by the isobologram and combination-index methods of Chou and Talalay. Mechanistic studies addressed incorporation of troxacitabine into DNA and intracellular levels of troxacitabine and gemcitabine metabolites. For *in vivo *studies, we evaluated the effect of both drugs, alone and in combination, on the growth of established human pancreatic (AsPC-1) tumors implanted subcutaneously in nude mice. Statistical analysis was calculated by a one-way ANOVA with Dunnett as a post-test and the two-tailed unpaired *t *test using GraphPad prism software.

**Results:**

Synergy, evaluated using the CalcuSyn Software, was observed in all four cell-lines at multiple drug concentrations resulting in combination indices under 0.7 at Fa of 0.5 (50% reduction of cell growth). The effects of drug exposures on troxacitabine and gemcitabine nucleotide pools were analyzed, and although gemcitabine reduced phosphorylation of troxacitabine when cells were exposed at equal drug concentrations, there was no effect on phosphorylated pools at drug combinations that were synergistic. The amount of troxacitabine incorporated into DNA was also not affected by the presence of gemcitabine. *In vivo *testing against a human pancreatic (AsPC-1) xenograft mouse tumor model indicated that both drugs were more than additive at well-tolerated doses and schedule. The biological basis for this synergy is unclear as we did not observe changes in apoptosis, DNA repair, troxacitabine incorporation into DNA or troxacitabine metabolism in the presence of gemcitabine.

**Conclusion:**

These data, together with phase I clinical data showing tolerability of both agents when combined, suggest combination therapy with troxacitabine and gemcitabine warrants further evaluation in advanced pancreatic cancer patients.

## Background

Pancreatic adenocarcinoma is one of the leading causes of cancer death with mortality rates almost identical to incidence rates [1]. Diagnosis usually occurs at late stages, making surgical intervention almost unfeasible due to low survival rates [2]. 5-Fluorouracil (5-FU), a previous standard treatment for advanced pancreatic adenocarcinoma, had a modest response rate of 20% and achieved median survivals of only 2–6 months [3]. Gemcitabine (GEMZAR; Eli Lilly), a cell-cycle specific inhibitor of DNA synthesis and ribonucleotide reductase, has been directly compared to 5-FU in a randomized phase III study in advanced adenocarcinoma of the pancreas, in which gemcitabine improved quality of life and extended survival by two months [4]. This study led to the approval by the U.S. Food and Drug Administration for gemcitabine as first-line treatment for patients with locally advanced or metastatic cancer of the pancreas. It is clear that novel therapeutic agents and/or combinations are needed for the treatment of pancreatic cancer.

Troxacitabine (Troxatyl™; Shire Biochem, Inc., exclusively licensed to SGX Pharmaceuticals, Inc.), like gemcitabine, is a deoxycytidine nucleoside analog. Preclinical studies demonstrated that it has broad and potent antitumor efficacy against both solid and haematological human tumor xenografts [5-9]. Moreover, troxacitabine was shown to be active against two human pancreatic cancer cell lines, Panc-1 and MIA PaCa-2, grown as xenografts in nude mice [10]. In these studies, troxacitabine exhibited greater reduction in tumor size compared to gemcitabine in Panc-1 xenografts and equivalent activity in MIA PaCa-2 xenografts.

Troxacitabine, which has an unnatural β-L-configuration, has different mechanistic properties compared to cytarabine and gemcitabine. Troxacitabine is not a permeant for nucleoside transporters [11], is resistant to deamination [5,7], does not inhibit ribonucleotide reductase and is phosphorylated to its triphosphate by 3-phosphoglycerate kinase instead of nucleoside diphosphate kinase [12-14]. In contrast to most other nucleoside analogs, intracellular accumulation of phosphorylated metabolites of troxacitabine is proportional to its extracellular concentrations [15]. In addition, inefficient removal of troxacitabine within DNA by 3'→5' exonucleases may result in prolonged retention of troxacitabine, leading to its cytotoxicity [16,17].

Combination therapy is a major strategy for overcoming drug resistance and improving responses and cure rates. In general, agents that have distinct and complementary biochemical mechanisms of action are exploited for possible biochemical synergy. The rationale for studying troxacitabine and gemcitabine, although structurally closely related and both targeting DNA, in combination regimens is provided by differences in their activation and elimination pathways. Combining two nucleoside analogs, although a new paradigm in oncology, is a standard strategy in virology. Complementary antineoplastic activity has been documented for troxacitabine and cytarabine [18] as well as for gemcitabine and cytarabine [19]. Because troxacitabine has potent antitumor activity in human pancreatic xenografts [10] and gemcitabine is currently used as first-line treatment for patients with locally advanced or metastatic cancer of the pancreas, the current work was undertaken to determine if they can be used concomitantly, thereby providing additive or synergistic benefits against pancreatic cancer. We thus evaluated the growth inhibitory potential of troxacitabine, gemcitabine, and their combination in a panel of human pancreatic cancer cell lines and on their *in vivo *antitumor efficacy against a human pancreatic cancer (AsPC-1) xenograft.

## Methods

### Materials

Troxacitabine (Troxatyl™, (-)-2'-deoxy-3'-oxacytidine; Mr = 213) was synthesized at Shire Biochem [20] and [^3^H]troxacitabine (3.9 Ci/mmol) was prepared by Moravek Biochemicals (Brea, CA). Gemcitabine hydrochloride for injection (GEMZAR, Eli Lilly Canada Inc.; 1 g per vial) was purchased from the Montreal General Hospital (Montreal, Canada). [5,6 ^3^H]Uridine (specific activity of 41.2 Ci/mmol) was obtained from Moravek Biochemicals (Brea, CA) with a purity of >97% as confirmed by high performance liquid chromatography (HPLC). All other reagents used were of analytical grade and were obtained from commercial sources. Tissue culture (12-well) plates, tissue culture medium Roswell Park Memorial Institute (RPMI) 1640 medium, Dulbecco's Minimal Eagle's Medium (DMEM), McCoy's 5A Medium, and fetal bovine serum were purchased from Gibco BRL (Burlington, ON, Canada).

### Growth and maintenance of cell lines

Human pancreatic adenocarcinoma cell lines, Panc-1, Capan-2, MIA PaCa-2, and AsPC-1 were purchased from the American Type Culture Collection (Manassas, VA.). Stock cell lines, which were demonstrated to be free of *Mycoplasma *by PCR analysis (Stratagene, LaJolla, CA), were routinely cultured in DMEM (Panc-1 and MIA PaCa-2), RPMI (AsPC-1) or McCoy's 5A (Capan-2) with 10% fetal bovine serum and maintained as adherent cultures at 37°C in a humidified atmosphere containing 5% CO_2_. Glutamine, HEPES or sodium pyruvate supplements were added to maintain proper cell growth according to the instructions from the American Type Culture Collection.

### Growth inhibition studies

Exponentially growing cells were seeded in 12-well plates at densities of 4 × 10^4 ^(MIA PaCa-2), 3 × 10^4 ^(Panc-1), and 8 × 10^4 ^(Capan-2 and AsPc-1) cells/well and allowed to attach for 24 h. Cells were exposed to graded concentrations (three replicates) of troxacitabine or gemcitabine either alone or in combination for 72 h. A total of eight drug concentrations (10^-3 ^to 10^-10 ^M for troxacitabine and 10^-5 ^to 10^-12 ^M for gemcitabine) (T) plus a drug-free control (C) and a time zero (T_0_, time at which test compound was added) were evaluated. At time zero and at the end of the 72 h treatment, cells were harvested by trypsinization and counted using electronic particle counting (Coulter Electronics Inc., Luton, UK). Chemosensitivity values were expressed as the drug concentration that inhibited cell growth by 50% (GI_50_) [calculated from [(T-T_0_)/(C-T_0_)] × 100 = 50, which is the drug concentration resulting in a 50% reduction in the net cell number of drug-treated cultures relative to the increase in control cultures during the drug incubation period] determined from concentration-effect relationships using GraphPad prism 2.01 (GraphPad Software, San Diego, CA).

### Analysis of combined drug effects

Drug synergy was determined by the isobologram and combination-index methods, derived from the median-effect principle of Chou and Talalay [21] using the CalcuSyn software (Biosoft, Ferguson, MO). Data obtained from the growth inhibitory experiments were used to perform these analyses. The isobologram method is a graphical representation of the pharmacologic interaction and is formed by selecting a desired fractional cell kill (Fa) and plotting the individual drug doses required to generate that Fa on their respective x- and y-axes. A straight line is then drawn to connect the points. The observed dose combination of the two agents that achieved that particular Fa is then plotted on the isobologram. Combination data points that fall on the line represent an additive drug-drug interaction, whereas data points that fall below or above the line represent synergism or antagonism, respectively. The combination-index (CI) method is a mathematical and quantitative representation of a two-drug pharmacologic interaction. Using data from the growth inhibitory experiments and computerized software, CI values are generated over a range of Fa levels from 0.05 – 0.90 (5% – 90% growth inhibition). A CI of 1 indicates an additive effect between two agents, whereas a CI < 1 or CI > 1 indicates synergism or antagonism, respectively.

### Cellular uptake assays

Cellular uptake of 10 μM [^3^H]troxacitabine and [^3^H]uridine by Capan-2, AsPC-1, MIA PaCa-2 and Panc-1 cells was measured for four hours in the absence or presence of excess unlabeled nucleoside. At the end of incubation periods, cells were washed free of [^3^H] permeant and solubilised to measure cell-associated radioactivity as described previously [11]. The uptake of uridine, a known high-activity permeant for both equilibrative and concentrative nucleoside transporters [22], was examined and compared to that of troxacitabine, which has previously been shown to be a non-permeant of the nucleoside transporters, entering cells primarily by passive diffusion [11]. Excess unlabeled troxacitabine or uridine was included since their presence blocks mediated uptake of radiolabeled nucleoside via nucleoside transporters. Uptake assays were conducted in 20 mM Tris-HCl buffer containing 3 mM K_2_HPO_4_, 1 mM MgCl_2_.6H_2_O, 2 mM CaCl_2_, 5 mM glucose, and 130 mM NaCl (pH 7.4); 300 ± 15 mOsm and at room temperature.

### Intracellular phosphorylated metabolites

Formation of tritiated metabolites of troxacitabine was determined in AsPC-1 cells. Logarithmically growing AsPC-1 cells (2 × 10^6 ^cells/flask) were incubated in the presence of [^3^H]troxacitabine alone or in combination with gemcitabine using equimolar drug concentrations (10 μM [^3^H]troxacitabine in the presence or absence of 10 μM non-radioactive gemcitabine) or 100:1 troxacitabine:gemcitabine drug concentrations (10 μM [^3^H]troxacitabine in the presence or absence of 0.1 μM gemcitabine) for 4 h. Formation of intracellular phosphorylated metabolites was also evaluated after 24-h exposures using lower drug concentrations (300 nM [^3^H]troxacitabine and 0.5 nM gemcitabine). At the end of incubation periods (all at 37°C), the medium was removed and the cells were trypsinized and washed by centrifugation with cold phosphate buffered saline. The cell pellets were snap frozen and stored at -80°C until analysis by HPLC.

Nucleotides were extracted from cell pellets with 10% trichloroacetic acid (TCA), and the extracts were neutralized with 1 N tri-n-octylamine in Freon as described previously [23]. The TCA insoluble pellet, representing the nucleotides incorporated into DNA, was re-solubilized in 0.5 N NaOH and radioactivity was measured with a Beckman LS5000TD scintillation counter and protein levels were determined with the Bradford protein assay (Bio-Rad Laboratories, Mississauga, Canada). For determination of mono-, di-, and triphosphates of troxacitabine and gemcitabine, HPLC with online radiodetection (Canberra Packard Canada Ltd., Montreal, Canada) and UV detection was used as described earlier [11]. Samples were analyzed with a Whatman 10 SAX (4.6 × 250 mm) column with a linear gradient of 10 mM ammonium phosphate (pH 2.9) to 750 mM ammonium phosphate (pH 4.1) at flow rates of 1.5 mL/min. HPLC analysis of 15 nmol each of troxacitabine, troxacitabine mono-, di- and triphosphates standards yielded retention times of 6.1, 10.4, 21.1 and 39.9 min, respectively. ATP, ADP, and AMP levels were also determined to monitor the quality of the extraction procedure [24].

### In vivo studies

*In vivo *antitumor efficacy studies were performed in the animal facility at Shire BioChem Inc. with the prior approval of the local Institutional Animal Care Committee and in agreement with the guidelines provided by Canadian Council for Animal Care. Female Fox Chase SCID^® ^mice, between five- and six-weeks old, were purchased from Charles Rivers Laboratories (St-Constant, Canada). Animals were maintained under specific pathogen-free conditions and provided with sterile food and water *ad libitum*. The mice were allowed to acclimate for at least five days. Mice were injected subcutaneously (SC) with 5 × 10^6 ^human pancreatic AsPC-1 tumor cells. Tumor-bearing animals were randomized (nine per group) and treatment was started when mean tumor volumes reached 145–150 mm^3 ^(Day 14). Troxacitabine was administered intravenously (IV) at 1 and 5 mg/kg q3d × 4 and gemcitabine was administered intraperitoneally (IP) at 80 mg/kg q3d × 4. In the combination studies, drugs were injected sequentially using the same regimens (troxacitabine first, followed by gemcitabine 1 h later; gemcitabine first, followed by troxacitabine 1 h later).

Tumor growth was followed every other day by measuring tumor length (L) and width (W) using a caliper. Each set of measurements was converted to tumor volume (*TV*; mm^3^) using the standard formula, *TV *= (L xW^2^)/2. The tumor volume at day *n *was expressed as the relative tumor volume (*RTV*) according to the following formula: *RTV *= *TVn */*TV*_0_, where *TVn *is the tumor volume at day *n *(day 45 in this study) and *TV*_0 _is the tumor volume at day 14 (time at which treatment begins). Drug efficacy was assessed at Day 45, the time at which animals from the control group had to be sacrificed due to tumor burden, as the percentage of T/C, determined by calculating *RVT *as: T/C% = 100 × (mean *RTV *of treated group)/(mean *RVT *of control group). According to the NCI standards, a % T/C ≤ 42 is indicative of antitumor activity [25-27]. Statistical analysis was calculated by a one-way ANOVA with Dunnett as a post-test [28] and the two-tailed unpaired *t *test using GraphPad prism, version 2.01 (GraphPad Software, San Diego, CA). Differences were considered to be significant at *P *< 0.05. Animals were weighed at least twice weekly during and after treatment until completion of the study. The mice were examined frequently for overt signs of any adverse drug-related side effects. Animals were euthanized if they showed more than 15% body weight loss for three consecutive days or >20% body weight loss during a single day.

## Results

### *In vitro *combination studies with troxacitabine and gemcitabine

**Figure 1:**
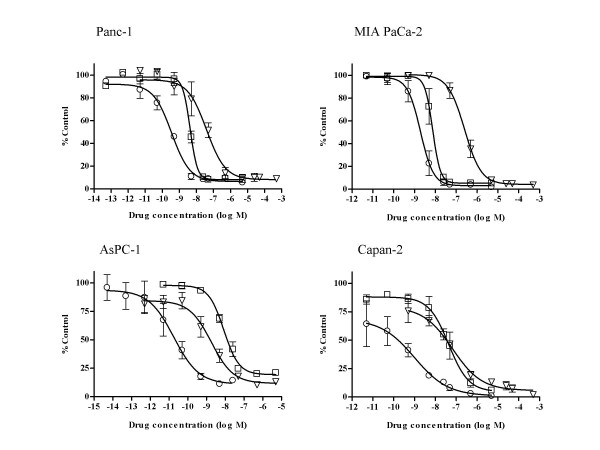
**Effect of troxacitabine and gemcitabine on growth of human pancreatic tumor cell lines**. Panc-1, MIA PaCa-2, AsPc-1 and Capan-2 cells were exposed to graded concentrations of troxacitabine or gemcitabine either alone or in combination at a ratio of 1:100 of gemcitabine vs. troxacitabine, for 72 h, after which cells were harvested by trypsinization and their numbers determined using electronic particle counting. Each data point represents the mean ± SD of three determinations. Gemcitabine (open squares), troxacitabine (open inverted triangle), gemcitabine + troxacitabine (open circle). The GI_50 _values for exposures to troxacitabine and gemcitabine alone are given in Table 2.

**Figure 2:**
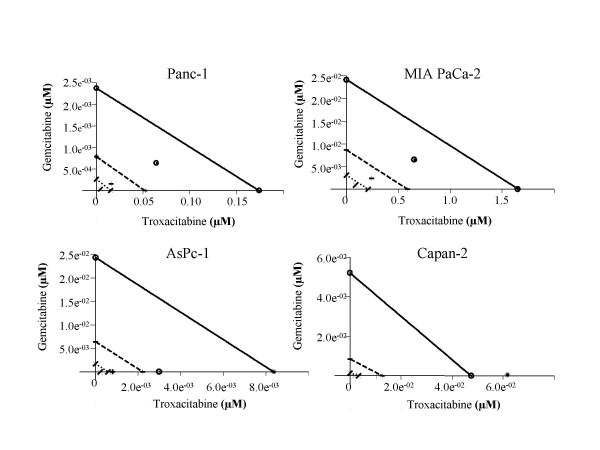
**Isobolograms of *in vitro *drug combinations**. Isobologram analysis of the combination of gemcitabine and troxacitabine in Panc-1, MIA PaCa-2, AsPc-1 and Capan-2 cells. The individual doses of gemcitabine and troxacitabine to achieve 90% (straight line) growth inhibition (Fa = 0.90), 75% (hyphenated line) growth inhibition (Fa = 0.75), and 50% (dotted line) growth inhibition (Fa = 0.50) were plotted on the x- and y-axes. Combination index (CI) values calculated using Calcusyn software is represented by points above (indicate antagonism between drugs) or below the lines (indicate synergy). (X symbol) ED_50, _(plus sign) ED_75 _and (open dotted circle ) ED_90_.

The four human pancreatic cancer cell lines (AsPC-1, Capan-2, MIA PaCa-2, and Panc-1) were seeded in 12-well plates, allowed to attach for 24 h and exposed to graded concentrations of troxacitabine and gemcitabine, either alone or in combination for 72 h (Fig. 1). Since these growth inhibitory studies demonstrated that gemcitabine was more potent than troxacitabine in three of the four cell lines (summarized in Table 1), combination studies were done using a drug ratio of 1:100 (gemcitabine: troxacitabine) over the range of drug concentrations tested. Combination treatments yielded significantly greater growth inhibition than either agent alone in all cell lines tested (Fig. 1). The isobologram and combination-index methods developed by Chou and Talalay [21] were used to confirm and quantify the synergism observed with gemcitabine and troxacitabine. Isobolograms were constructed for Fa values of 0.50, 0.75, and 0.90, representing 50%, 75% and 90% growth inhibition, respectively (Fig. 2). The CI values at Fa of 0.5 were calculated using CalcuSyn software and are summarized in Table 1. These results indicated that gemcitabine and troxacitabine were synergistic with CI values of 0.41, 0.71, 0.37, and 0.52, respectively, for Panc-1, MIA PaCa-2, AsPC-1 and Capan-2 cells. Both methods indicated synergism across a broad range of concentrations in all four pancreatic tumor cell lines.

**Table 1:** Growth inhibition evaluation of troxacitabine and gemcitabine in four pancreatic cell lines.

**Cell Line**	**GI_50 _(nM) ± SEM**		**CI**^a^
		
	**Gemcitabine**	**Troxacitabine**	
Panc-1	4 ± 0.7	40 ± 10	0.41
MIA PaCa-2	13 ± 3	330 ± 20	0.71
AsPC-1	10 ± 2	4 ± 1	0.37
Capan-2	70 ± 20	150 ± 30	0.52

^a ^Fa denotes fraction affected (e.g., Fa of 0.5 is equivalent to a 50% reduction in cell growth). CI >1 denotes antagonism, CI = 1 denotes additivity, and CI < 1 denotes synergism.

Cells were exposed to graded concentrations of troxacitabine or gemcitabine either alone or in combination at a ratio of 1:100 gemcitabine vs. troxacitabine for 72 h as shown in Fig. 1. Values for GI_50 _(± SEM) (the drug concentration that inhibited cell growth by 50% as determined from concentration-effect relationships using GraphPad Prism version 3.0 software) from three experiments, each conducted with three replicates, were calculated from non-linear regression analysis of data plotted as percentages of control values against the logarithm of drug concentrations. CI (combination index) values at a Fa value of 0.5 were generated from the data of Fig. 2 with the CalcuSyn, version 2.0 software.

**Table 2:** Uptake of 10 μM [^3^H]troxacitabine and 10 μM [^3^H]uridine in four pancreatic adenocarcinoma cell lines.

**Cell lines**	**[^3^H]Troxacitabine**	**[^3^H]Troxacitabine + 1 mM cold troxacitabine**	**[^3^H]Uridine**	**[^3^H]Uridine + 1 mM cold Uridine **
**Capan-2**	10.2 ± 0.2	10.4 ± 0.1	1028 ± 74	65 ± 6
**AsPc-1**	19.3 ± 1.5	18.5 ± 1.4	6337 ± 183	326 ± 33
**MIA PaCa-2**	8.3 ± 0.3	8.2 ± 0.9	3070 ± 244	157 ± 1
**Panc-1**	12.7 ± 0.8	10.0 ± 0.7	2714 ± 302	158 ± 2

Comparative uptake (4 h) of 10 μM [^3^H]troxacitabine and 10 μM [^3^H]uridine in four pancreatic adenocarcinoma cell lines in the absence or presence of excess non-radioactive (cold) nucleoside. Each value (pmol/10^6 ^cells) represents the mean (± SD) of three determinations.

### Comparison of uptake of [^3^H]troxacitabine and [^3^H]uridine in pancreatic adenocarcinoma cell lines

The uptake of 10 μM [^3^H]troxacitabine in Capan-2, AsPC-1, MIA PaCa-2 and Panc-1 cells was measured at 4-h time intervals in the absence and presence of excess (100-fold) non-radioactive troxacitabine. The uptake of 10 μM [^3^H]uridine in the absence and presence of excess (100-fold) non-radioactive uridine to block the transport and subsequent metabolism steps of the uptake process was included as a control for comparison; uridine was used rather than deoxycytidine, which is structurally more closely related to troxacitabine, because of its higher activity as a permeant for nucleoside transporters [22]. Relative accumulation values (pmol/10^6 ^cells) of troxacitabine and uridine in AsPC-1, MIA PaCa-2, Capan-2 and Panc-1 cells are summarized in Table 2. All four cell lines exhibited a large difference in capacity for uptake of 10 μM [^3^H]troxacitabine and [^3^H]uridine, with 100 to 400-fold greater uptake observed for uridine than for troxacitabine. The presence of a high concentration (100-fold) of non-radioactive uridine inhibited uptake of 10 μM [^3^H]uridine in all four cell lines by >90%, indicating that uptake, which included the transport step of the uptake process, was mediated. In contrast, the presence of high concentrations (100-fold) of non-radioactive troxacitabine had no effect on the uptake of 10 μM [^3^H]troxacitabine by any of the cell lines, suggesting that uptake (and subsequent metabolism) of troxacitabine was slow, with the permeation step occurring by passive diffusion and/or a low-affinity transport process, consistent with our previous findings [11].

### Analyses of phosphorylated metabolites of troxacitabine

To understand the biological effects of troxacitabine and gemcitabine either alone or in combination, [^3^H]troxacitabine metabolites were analyzed under different incubation conditions as described in Materials and Methods. The AsPC-1 cell line was chosen for these studies since it had (i) the highest uptake and accumulation of [^3^H]troxacitabine among the four cell lines, and (ii) a good synergy response. When cells were exposed to equal concentrations (10 μM) of [^3^H]troxacitabine and gemcitabine, all three troxacitabine phosphorylated species were reduced by 50% (Table 3). When cells were exposed to 10 μM [^3^H]troxacitabine in the presence of 0.1 μM gemcitabine, while there was reduction in troxacitabine monophosphate, there was no effect on the di- and tri-phosphorylated species. No reduction in phosphorylated metabolites of troxacitabine were observed in AsPC-1 cells exposed to 300 nM [^3^H]troxacitabine and 0.5 nM gemcitabine for 24 h. We did not observe, in these three different conditions, changes in troxacitabine incorporation into DNA in the presence of gemcitabine. When similar experiments were conducted using [^3^H]gemcitabine to monitor its metabolites in the presence or absence of troxacitabine, no changes were observed in pools of phosphorylated gemcitabine metabolites (data not shown). These results demonstrated that the synergies observed during *in vitro *combination treatments were not caused by changes in the relative proportions of phosphorylated metabolites of troxacitabine and gemcitabine or by an increase in troxacitabine incorporation into DNA.

**Table 3:** HPLC analysis of troxacitabine metabolites after exposure to gemcitabine.

**Treatment**	**Troxacitabine incorporation ** **into DNA** **(CPM/10^6 ^cells)**	**Metabolites** **(pmol/mg protein)**		
		**MP**	**DP**	**TP**
		
[^3^H]Troxacitabine 10 μM [^3^H]Troxacitabine 10 μM + Gemcitabine 10 μM	1248 ± 153 1226 ± 91	5.0 ± 1.6 2.8 ± 0.9	26.0 ± 7.0 13.0 ± 4.0	6.4 ± 1.5 3.2 ± 1.1
[^3^H]Troxacitabine 10 μM [^3^H]Troxacitabine 10 μM + Gemcitabine 0.1 μM	1248 ± 153 1097 ± 298	5.6 ± 1.5 1.7 ± 2.0	22.0 ± 5.0 23.0 ± 2.0	4.5 ± 1.7 5.2 ± 1.0
[^3^H]Troxacitabine 300 nM [^3^H]Troxacitabine 300 nM + Gemcitabine 0.5 nM	4039 ± 118 3838 ± 509	3.0 ± 0.1 2.0 ± 0.2	6.0 ± 0.2 5.9 ± 0.2	2.6 ± 0.3 2.5 ± 0.2

AsPc-1 cells were exposed to [^3^H]troxacitabine in absence or presence of gemcitabine as described in Materials and Methods. Cells were harvested by trypsinization, washed in cold PBS and extracted with 10% TCA for 10 min on ice. The acid-insoluble pellets, containing DNA into which [^3^H]troxacitabine was incorporated, were solubilized in 0.5 N NaOH and their radioactive content was determined. Values are presented as (CPM/10^6 ^cells ± SD). The supernatants, containing phosphorylated metabolites of [^3^H]troxacitabine, were analyzed by HPLC. Values (pmol/mg protein ± SEM) for troxacitabine monophosphate (MP), diphosphate (DP) and triphosphate (TP) are given below.

### *In vivo *antitumor efficacy studies

**Figure 3:**
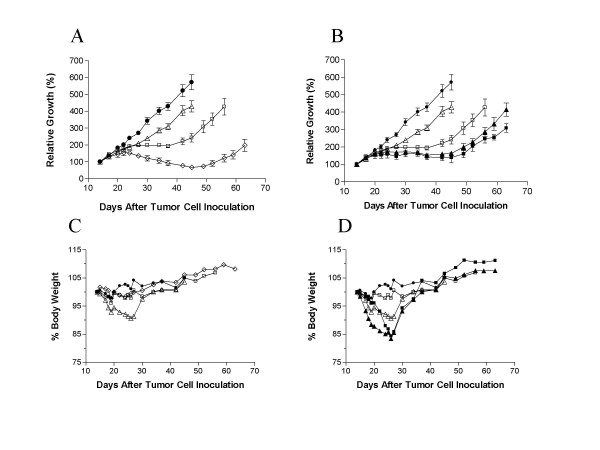
**Antitumor activity of troxacitabine and gemcitabine alone (A) or in combination (B) against human pancreatic cancer (AsPc-1) xenografts.**  Female SCID mice were inoculated SC with 5 x 10^6^ AsPc-1 cells (Day 0).  Treatment, which was initiated when tumors were established (Day 14), was given on Days 14, 17, 20, and 23.  Troxacitabine was given IV at 1 mg/kg (open square) and 5 mg/kg (open losange).  Gemcitabine (open triangle) was administered IP at 80 mg/kg.  The 1 mg/kg dose of troxacitabine was used for combination studies and drugs were given sequentially, 1h apart: gemcitabine followed by troxacitabine (closed triangle); troxacitabine followed by gemcitabine (closed square).  Saline control group (closed circle) was given IP at 5 mL/kg (Days 14, 17, 20, and 23).

The effects of the combination of troxacitabine and gemcitabine on human pancreatic AsPC-1 tumor xenografts were studied to determine if the synergy observed *in vitro *between troxacitabine and gemcitabine also occurred *in vivo*. Treatment with either troxacitabine (1 and 5 mg/kg q3d × 4) or gemcitabine (80 mg/kg q3d × 4) was initiated when tumors were established (mean tumor volumes of 145 to 150 mm^3^). Treatment was on Days 14, 17, 20, and 23. Sub-optimal doses of troxacitabine were given to enhance the likelihood of seeing synergism. Interestingly, the 5 mg/kg troxacitabine dose, which was well below the maximum tolerated dose [5,29,30], resulted in tumor regressions occurring from Day 37 till Day 52 with mean tumor volumes of 133 mm^3 ^to 97 mm^3 ^(*P *< 0.001). The % T/C for that group calculated at Day 45 (the time at which control mice were sacrificed because of tumor burden; Fig 3A) was 12%. No cures were observed and tumors regrew, reaching a mean of ~300 mm^3 ^at Day 65 (when the experiment was terminated). While the dose of 1 mg/kg was less active than 5 mg/kg, it also exhibited significant antitumor activity (T/C of 41% at Day 45, *P *= 0.0126), although no tumor regressions were observed. While gemcitabine was administered at a dose and schedule reported to give optimal antitumor efficacy in other pancreatic tumor models [10], it did not result in significant antitumor efficacy in the AsPC-1 pancreatic tumor model (T/C = 79% at Day 45, *P *= 0.31).

For the combination studies, we used the lower dose of troxacitabine (1 mg/kg) since we wanted to improve the antitumor activity of gemcitabine. Regardless of the sequence of administration, troxacitabine and gemcitabine had only slight synergistic effects (Fig. 3B and Table 4). Indeed, the observed T/C at Day 45 was 27% when troxacitabine was administered either one hour prior or one hour after gemcitabine administration. The observed T/C of 27% at Day 45 was slightly lower than the expected T/C of 32% (calculated by multiplying the %T/C of each treatment alone, that is 79% and 41% for gemcitabine and troxacitabine, respectively). The *P *value for the troxacitabine + gemcitabine combination group was 0.0021 when compared with the saline-treated animals, and 0.0074 when compared with the gemcitabine-treated animals. These results indicated that both drugs were slightly more than additive when combined. While troxacitabine administered as a single agent at 1 and 5 mg/kg did not result in body weight loss, gemcitabine treatment (80 mg/kg q3d × 4) resulted in ~8 % body weight loss from Days 18–27 (Fig 3C). Combination treatments resulted in up to 15 % body weight loss from Days 24–27 (Fig 3D). In all groups, body weight increases were observed after treatment stopped, indicating that the doses and schedule used were well tolerated.

**Table 4:** Troxacitabine and gemcitabine combination studies in the human pancreatic AsPc-1 xenograft tumor model.

**Group**	**Dose and Schedule**	**Tumor Volumes mm^3 ^Day 45**	**% T/C**^a^	***P *values** **Compared with saline control group**	***P *values** **Compared with gemcitabine treated group**
Control (Saline)	5 mL/kg IV q3d × 4	804	-		
Troxacitabine	1 mg/kg IV q3d × 4	324	41%	*P *= 0.0126	
Troxacitabine	5 mg/kg IV q3d × 4	90	12%	*P *< 0.001	
Gemcitabine	80 mg/kg IP q3d × 4	631	79%	*P *= 0.31	
Troxacitabine + Gemcitabine	1 mg/kg IV + 80 mg/kg IP q3d × 4	215	27%	*P *= 0.0021	*P *= 0.0074

^a ^Tumor measurements were taken twice weekly. Drug efficacy was assessed at Day 45, time at which animals from the control group had to be sacrificed due to tumor burden, as the percentage of T/C, determined by calculating RVT as: T/C% = 100 × (mean RTV of treated group)/(mean RVT of control group).

Female SCID mice were inoculated SC with 5 × 10^6 ^AsPc-1 cells (Day 0). Treatment with troxacitabine and/or gemcitabine was started 14 days after tumor cell inoculation, once the mice had developed palpable tumors.

## Discussion

Monotherapy of pancreatic cancer with gemcitabine results in only a marginal survival benefit [3]. Moreover, we have previously shown that troxacitabine had activity in human pancreatic cancers both pre-clinically [10] and clinically [31]. Although both compounds are deoxycytidine nucleoside analogs, troxacitabine is mechanistically different from gemcitabine (reviewed in references [29,30]). Its pharmacokinetics are different from those of most D-nucleoside analogs – e.g., it has an unusually long terminal half-life (82 h) [32]. Combining two nucleoside analogs, although a new paradigm in oncology, is a standard strategy in virology. Because troxacitabine is unique among anticancer nucleoside drugs in terms of its structure, pharmacokinetics, intracellular transport, metabolism, and mechanism of action, the current study asked if troxacitabine and gemcitabine acted synergistically in pancreatic cancer cell lines and a human pancreatic xenograft mouse tumor model.

Troxacitabine and gemcitabine demonstrated synergistic activity *in vitro *in the four human pancreatic cell lines tested. Because troxacitabine and gemcitabine both undergo a series of phosphorylation reactions through a first rate-limiting step catalyzed by deoxycytidine kinase (dCK) to form the active triphosphate nucleotide, we examined if their combined administration resulted in changes in their respective phosphorylated metabolites. Since troxacitabine triphosphate is a poor feedback inhibitor of dCK [18], it is not likely to limit accumulation of gemcitabine triphosphate inside cells, and, as expected, no changes were observed in phosphorylated [^3^H]gemcitabine metabolites in troxacitabine-treated cells (data not shown). The converse was also true, in that the presence of gemcitabine did not affect the levels of phosphorylated [^3^H]troxacitabine metabolites. These results indicated that changes in phosphorylated metabolites of either troxacitabine or gemcitabine were not responsible for the synergy observed by combination treatment. A positive interaction between gemcitabine and other nucleoside analogs have been observed in several previous studies [18,19,33,34]. Although the molecular mechanism of potentiation in these studies has been attributed to an effect on DNA damage, we did not observe differences in troxacitabine incorporation into DNA or formation of phosphorylated metabolites in the presence of gemcitabine. The biological basis of this synergy remains unclear; DNA repair and apoptosis were unchanged by the combination [35] and thus, we cannot explain the observed synergy.

Experiments were conducted to determine if the synergy observed in cultured cell lines when exposed simultaneously to gemcitabine and troxacitabine also occurred in an *in vivo *human xenograft model of AsPC-1 cells in SCID mice. The data indicated that, regardless of the sequence of administration, both troxacitabine and gemcitabine had greater than additive effects. Thus, the synergy observed *in vitro *was extended to *in vivo *antitumor efficacy studies at well-tolerated doses and schedules. The results presented here led to clinical trials of combination therapy with troxacitabine and gemcitabine in pancreatic cancer patients. A dose-escalation phase I study of troxacitabine and gemcitabine was well-tolerated with doses currently approaching the single-agent mean tolerated doses (i.e., 8 mg/m^2 ^troxacitabine on Day 1 with 1,000 mg/m^2 ^gemcitabine on Day 1 and 8 of a 3-week cycle) [36].

## Conclusion

In conclusion, these observations, combined with phase II studies in which troxacitabine was evaluated as first-line therapy in 54 patients and gave comparable overall results to those reported for gemcitabine in randomized trials [31], support further evaluation of troxacitabine in combination with gemcitabine in patients with advanced pancreatic cancer.

## Abbreviations

Adenosine 5' triphosphate (ATP); Adenosine 5' diphosphate; Adenosine 5' monophosphate (AMP); Combination Index (CI); Deoxycytidine kinase (dCK); Every three days with a total of 4 administrations (q3d × 4); 5-Fluorouracil (5-FU), High Performance Liquid Chromatography (HPLC), Intraperitoneally (IP), Intravenously (IV), Severe Combined Immune Deficient Mice (SCID^®^); Subcutaneously (SC); Standard deviation (SD); Standard error of the mean (SEM); Treated vs. Control (T/C).

## Competing interests

The author(s) declare that they have no competing interests.

## Authors' contributions

HG was the PI at Shire BioChem and was responsible for study design, interpretation of the data and revision of the manuscript.

VLD was responsible for HPLC data acquisition and analysis of the work presented and the preparation of the manuscript.

DYB and LL coordinated and supervised the *in vivo *studies.

CEC and JRM coordinated and supervised the growth inhibition and transport studies.

MG is responsible for troxacitabine activities.

CKW and MLC participated in the study design and analysis of the growth inhibtion and transport data.

All authors read and approved the final manuscript.

## Pre-publication history

The pre-publication history for this paper can be accessed here:


